# Heritable and measured polygenic influences on Mild Cognitive Impairment (MCI) in the Vietnam Era Twin Study of Aging (VETSA)

**DOI:** 10.1002/alz70857_104205

**Published:** 2025-12-25

**Authors:** Chandra A. Reynolds, Nathan A. Gillespie, Michael C. Neale, Jeremy A. Elman, Christine Fennema‐Notestine, Carol E. Franz, Michael J. Lyons, Matthew S. Panizzon, William S. Kremen

**Affiliations:** ^1^ University of Colorado Boulder, Boulder, CO, USA; ^2^ Virginia Commonwealth Univeristy, Richmond, VA, USA; ^3^ University of California San Diego, La Jolla, CA, USA; ^4^ Boston University, Boston, MA, USA

## Abstract

**Background:**

Mild Cognitive Impairment (MCI) precedes Alzheimer's disease (AD) and may represent an early stage of pathology. The heritability of AD is about 58‐71% compared to midlife MCI at 40‐48%. We followed individuals into later life, expecting that heritability for MCI will be higher due to a longer assessment window, and by considering stability of diagnosis over time. In addition, we evaluated the contributions of polygenic scores (PGS) for AD and educational attainment in a longitudinal twin cohort.

**Method:**

MCI diagnoses were available for 1593 twins (1288 from complete pairs) from the Vietnam Era Twin Study of Aging (VETSA), a population‐based cohort of male twins with up to 4 longitudinal assessments (ages 52.1 to 78.5 years). MCI status was assessed using Jak/Bondi criteria where impairments 1.5 SD below the mean on 2 or more tests within a domain met criteria, after adjusting for young adult cognitive ability. Individuals whose MCI diagnoses were unstable longitudinally (8.7%; “reverters”) were treated as unaffected in analyses. Biometrical analyses controlled for age, and PGS adjusted for 10 ancestry PCs, were modeled as contributors to heritability. Amnestic (aMCI) and non‐amnestic MCI (naMCI) subtypes were considered.

**Result:**

Altogether 18.0% developed MCI during follow‐up (aMCI 11.9%, naMCI 6.1%). The average age at diagnosis was 63.8 years (SD=7.0). Biometrical analyses of MCI diagnoses (382 monozygotic, 262 dizygotic pairs) suggested heritability of general MCI was 42.1% (CI=22.2%, 59.3%) and for aMCI heritability was 57.9% (CI=36.6%, 74.3%). Remaining contributions were associated with person‐specific environmental influences. Heritability of naMCI was not estimable given limited concordant pairs. Removing reverters increased heritability (i.e., total MCI=46.9%, CI=26.8%, 63.8%; aMCI=61.1%, CI=39.9%, 77.1%). AD and education PGSs were not significantly associated with general or aMCI risk, contributing < 1%.

**Conclusion:**

Our results suggest that with follow‐up into later life and considering stability of diagnosis, heritability for amnestic MCI is on par with heritability estimates for AD, while for general MCI estimates were lower, although confidence intervals overlapped. Despite strong heritability, genetic contributions to amnestic and total MCI are not captured by extant polygenic scores for AD or educational attainment warranting additional follow‐up.

